# The Hidden Perils of Allopurinol: A Systematic Review of Allopurinol-Induced DRESS (Drug Reaction With Eosinophilia and Systemic Symptoms) Syndrome

**DOI:** 10.7759/cureus.104474

**Published:** 2026-03-01

**Authors:** Areti Kalfoutzou, Christos Piperis, Pantelis Petroulakis, Adam Mylonakis

**Affiliations:** 1 Department of Medical Oncology, 251 Air Force General Hospital, Athens, GRC; 2 Department of Cardiology, Gennimatas General Hospital, Athens, GRC; 3 Second Department of Internal Medicine, 251 Air Force General Hospital, Athens, GRC; 4 First Department of Surgery, Laikon General Hospital, National and Kapodistrian University of Athens, Athens, GRC

**Keywords:** : allopurinol, blood eosinophilia, dress syndrome, drug hypersensitivity syndrome, systematic review

## Abstract

Drug hypersensitivity reactions are a clinically significant and potentially preventable cause of hospital admission, treatment interruption, and drug-related mortality worldwide. Within severe cutaneous adverse reactions (SCARs), a group that includes Stevens-Johnson syndrome/toxic epidermal necrolysis (SJS/TEN), acute generalized exanthematous pustulosis (AGEP), and drug reaction with eosinophilia and systemic symptoms (DRESS), DRESS stands out because of its delayed onset, multi-organ involvement, and frequent diagnostic uncertainty. Allopurinol is a commonly prescribed medication for hyperuricemia, but in rare cases, it can trigger DRESS. This systematic review was conducted to comprehensively summarize the demographic characteristics, clinical manifestations, diagnostic features, management strategies, and outcomes of reported cases of allopurinol-induced DRESS syndrome. Available case reports and case series were analyzed to consolidate patient characteristics, patterns of organ involvement, treatment approaches, and clinical outcomes. The findings indicate that allopurinol-induced DRESS most often presents with cutaneous manifestations and systemic involvement, most commonly affecting the liver and kidneys, and is associated with considerable morbidity and mortality despite treatment. These results emphasize the importance of early recognition, prompt drug discontinuation, and cautious prescribing of allopurinol, particularly in high-risk populations.

## Introduction and background

Allopurinol is a xanthine oxidase inhibitor that directly targets the enzyme responsible for catalyzing the conversion of hypoxanthine to xanthine, which is subsequently converted to uric acid [1]. It is the primary therapy for gout, renal stones, and hyperuricemia, and remains among the most frequently prescribed medications worldwide [1]. While generally well tolerated, it is recognized as a potential trigger of a multi-organ reaction known as drug reaction with eosinophilia and systemic symptoms (DRESS) syndrome or drug-induced hypersensitivity syndrome (DIHS) [2,3]. DRESS syndrome is a type IV hypersensitivity reaction, typically developing within eight weeks of drug exposure [2,4]. Mild cases present with non-specific symptoms, including a generalized maculopapular rash, fever, and lymphadenopathy, whereas more severe cases involve vital organs, such as the kidneys or liver, potentially leading to long-term complications [5]. Drugs associated with this syndrome, apart from allopurinol, include anticonvulsants, sulphonamides, and nonsteroidal anti-inflammatory drugs (NSAIDs), among others [3,6].

Allopurinol-induced DRESS syndrome has a reported incidence of 0.4% [7,8]. It most commonly affects middle-aged adults, with a predominance in men [9]. Older adults with multiple comorbidities, particularly those with chronic kidney disease (CKD), are also at increased risk, possibly because reduced renal clearance leads to accumulation of oxypurinol, the active metabolite of allopurinol [10]. While the exact pathogenesis remains unclear, this syndrome has been associated with the presence of the HLA-B*58:01 haplotype, which is particularly prevalent among Asian populations, especially in individuals of Han Chinese descent [11,12]. An additional proposed mechanism involves immune dysregulation related to human herpesvirus 6 (HHV-6) reactivation, which is commonly identified during the diagnostic evaluation of these patients and has been reported in up to 60% of cases [13,14]. It remains uncertain whether viral infections alter drug metabolism to produce reactive metabolites or whether activation of drug-specific T cells subsequently triggers reactivation of latent viruses [13].

The RegiSCAR score has been widely used to aid in the diagnosis of DRESS syndrome by providing a set of clinical and laboratory criteria to classify the diagnosis as definite (score >5), probable (score 4 to 5), possible (score 2 to 3), or no DRESS (score <2) [3,15,16]. These criteria include key clinical features such as fever above 38 °C, enlarged lymph nodes at two or more sites, involvement of at least one internal organ, most commonly the liver or kidneys, and hematologic abnormalities such as eosinophilia, atypical lymphocytosis, or leukocytosis. Additionally, a skin eruption involving more than 50% of the body surface area, as well as persistence of symptoms after drug withdrawal, is taken into account [3]. The RegiSCAR scoring system helps clinicians differentiate DRESS from other drug hypersensitivity reactions and supports a more standardized approach to diagnosis in both clinical practice and research settings [17]. Other diagnostic tools, including Bocquet’s criteria, the Naranjo probability score, and the Singer and Wallace criteria, are now largely regarded as outdated or as having lower sensitivity and specificity compared with the RegiSCAR score [18].

Although allopurinol is one of the most frequently implicated drugs in DRESS, the evidence base is largely confined to isolated case reports and small case series, limiting clinicians’ ability to anticipate typical presentation patterns, latency, organ involvement, and outcomes. Therefore, this systematic review was undertaken to provide a comprehensive characterization of published cases of allopurinol-induced DRESS, including patient demographics, latency, clinical and laboratory features, patterns of organ involvement, diagnostic approaches, management strategies, and outcomes.

## Review

Materials and methods

Literature Search and Eligibility Criteria

Two independent reviewers conducted a systematic literature search in PubMed (n=232) and Scopus (n=56) for eligible studies published in English between January 1997 and 31 December 2024. The search included combinations of terms: "allopurinol", "DRESS syndrome", "Drug Reaction with Eosinophilia and Systemic Symptoms", "Drug-Induced Hypersensitivity Syndrome", and "DIHS". Additional studies were identified by searching the reference lists of relevant articles (n = 20), bringing the total to 308. Two reviewers independently screened titles and abstracts identified through the database search. Full-text articles of potentially eligible studies were subsequently assessed for inclusion. Disagreements were resolved through discussion and consultation with a senior investigator when necessary.

Inclusion criteria were limited to case reports and case series describing DRESS syndrome specifically caused by allopurinol. Exclusion criteria comprised duplicate publications, letters to the editor, systematic or narrative reviews, and case reports of DRESS syndrome induced by medications other than allopurinol. Cases in which patient outcomes were not documented were also excluded. Any disagreements between the reviewers were settled through consultation with a senior investigator.

**Figure 1:**
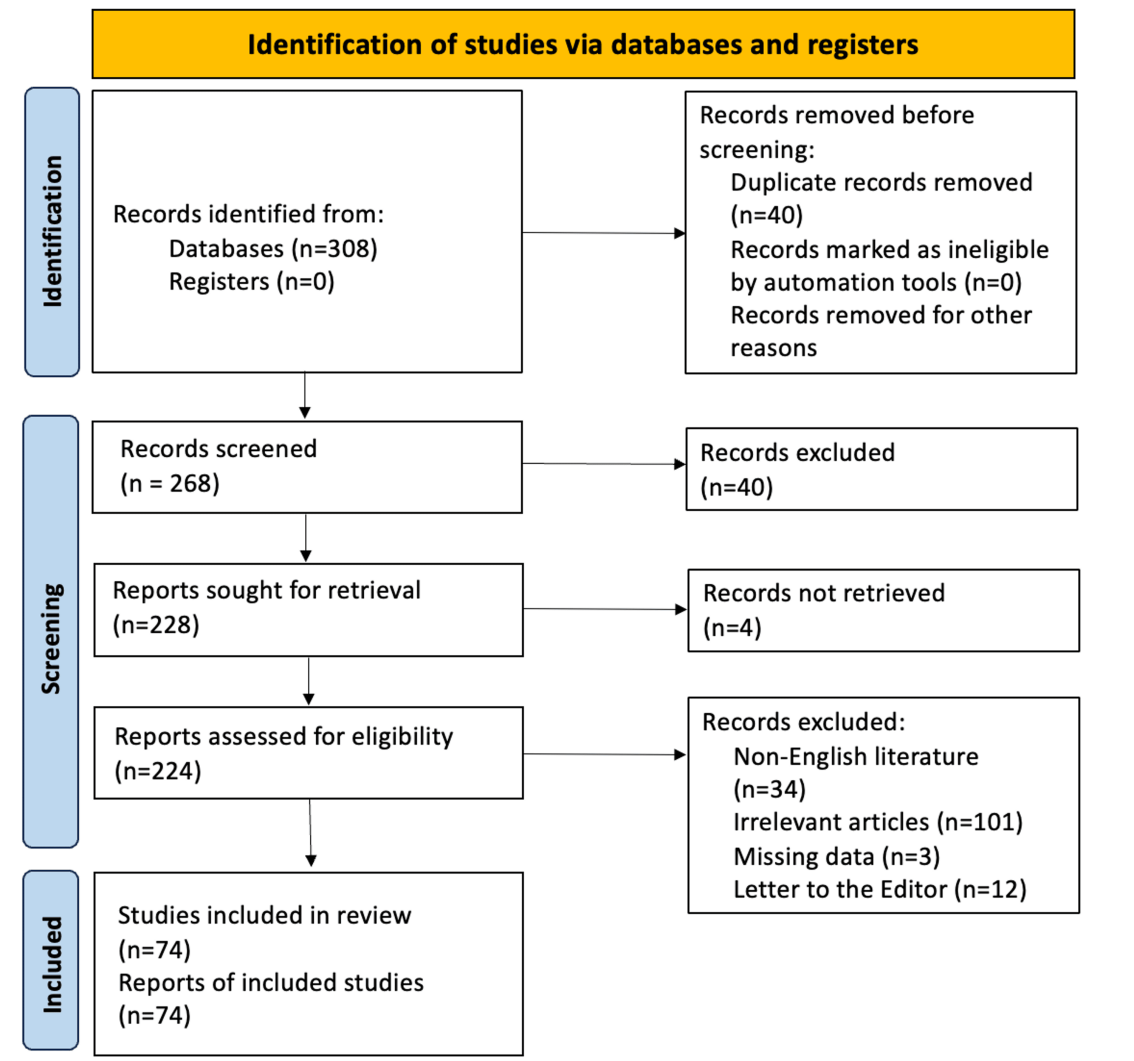
PRISMA 2020 flow diagram depicting the study selection process PRISMA: Preferred Reporting Items for Systematic Reviews and Meta-Analyses

The systematic review was conducted in accordance with the Preferred Reporting Items for Systematic Reviews and Meta-Analyses (PRISMA) guidelines [19], and the protocol was registered in the PROSPERO database (registration number CRD42024563499). Data extraction was carried out independently by two investigators using a predefined standardized data extraction form. The extracted variables included patient demographics, allopurinol dose and duration of therapy; clinical manifestations; organ and mucosal involvement; RegiSCAR score; laboratory findings; HLA B*58:01 haplotype status; biopsy findings; treatment strategies; clinical outcomes; and length of follow-up. The extracted data were cross-checked for accuracy before analysis, and any discrepancies were resolved by consensus. The literature search strategy and the PICO (Population, Intervention, Comparator, Outcomes) framework used to define the research question and eligibility criteria are summarized in Tables 1-2. The study selection process is illustrated in a PRISMA flow diagram (Figure 1).

**Table 1:** Databases searched, search terms, time frame, and language restrictions used for the identification of relevant studies on allopurinol-induced DRESS syndrome DRESS: drug reaction with eosinophilia and systemic symptoms

Database	Search terms	Time frame	Language
PubMED	“allopurinol” AND (“DRESS syndrome” OR “Drug Reaction with Eosinophilia and Systemic Symptoms” OR “Drug-Induced Hypersensitivity Syndrome” OR “DIHS”)	January 1997 – December 2024	English
SCOPUS			
Other sources	Reference lists of relevant articles		

**Table 2:** PICO framework defining the research question and eligibility criteria for the systematic review of allopurinol-induced DRESS syndrome PICO: Population, Intervention, Comparator, Outcomes; DRESS: drug reaction with eosinophilia and systemic symptoms

Component	Description
Population (P)	Patients of any age diagnosed with DRESS syndrome
Intervention/exposure (I)	Exposure to allopurinol
Comparator (C)	Not applicable (case reports and case series)
Outcome (O)	Clinical presentation, organ involvement, diagnostic methods, treatment strategies, complications, and patient outcomes (including mortality)

Data extraction and quality assessment

Data from the included case reports and case series were synthesized descriptively, with variables summarized as counts, ranges, and proportions based on the information reported in the original publications. Patients included in the case series were considered unique cases to estimate the variables of interest. Each domain was scored as “yes,” “no,” or “unclear,” and overall study quality was determined based on total scores. Due to the lack of some required information in several studies, rates were calculated based on the available data. Statistical analysis was conducted using IBM SPSS Statistics for Windows, Version 29.0 (IBM Corp, Armonk, NY). The quality assessment was conducted independently by two reviewers using the Joanna Briggs Institute (JBI) critical appraisal tool for case reports (Appendices). Disagreements were resolved through discussion until a consensus was reached.

Results

Study Selection

Data from 83 patients were retrieved from 74 studies for statistical analysis. The search strategy and study selection process are illustrated in the PRISMA flow chart (Figure 1). The methodological quality of the included studies, assessed using the Joanna Briggs Institute (JBI) critical appraisal checklist for case reports, was generally high. Most reports achieved the maximum score of 8/8 (58/83, 69.9%), followed by scores of 7/8 (22/83, 26.5%), and only three studies scored 6/8 (3.6%). The most frequently missing domains were related to incomplete reporting of diagnostic assessment details or adverse events. Detailed quality assessment results for individual studies are provided in the Appendices.

Demographics

Median age at diagnosis was 61 years (range: 13-88 years); three patients were in the pediatric-adolescent group [20-22], while 28 patients belonged to the elderly group (>65 years). The male-to-female ratio was 1.18:1 (46 males, 37 females). Patients' ethnicity was reported in 27 cases: 15 Asian, seven African-American, five Caucasian, and one Hispanic. Past medical history was reported in 64 cases, with 34 patients having two or more comorbidities apart from gout or hyperuricemia. The most frequent comorbidities reported were arterial hypertension (36 cases), CKD (28 cases), diabetes mellitus (19 cases), hyperlipidemia (nine cases), and coronary artery disease (CAD) (eight cases).

Genetic Associations

Genetic screening for the HLA-B*58:01 haplotype was performed in 19 cases and was positive in 16, including eight patients of Asian descent and three African-American patients. The indication for allopurinol prescription was specified in 67 cases, with 36 prescribed for hyperuricemia, 30 for gout, and two for leukemia in the context of chemotherapy initiation [23,24]. The dose of allopurinol was reported in 43 cases (mean: 236.04 mg, range: 100-1000 mg), whereas the time from allopurinol initiation to symptom onset was reported in 71 cases (median: 3.71 weeks, IQR: 2.68-5.00). Patients’ demographic characteristics are summarized in Table 3.

**Table 3:** Demographic details of patients with allopurinol-induced DRESS syndrome reported in the literature DRESS: drug reaction with eosinophilia and systemic symptoms

	No. of patients	Percentage (%)
Total no. of patients (n=83)		
Age group, years (median: 61 years; range: 13-88 years)		
10-20	3	3.61
21-30	2	2.40
31-40	9	10.84
41-50	11	13.25
51-65	30	36.14
>65	28	33.73
Sex (n=83)		
Male	46	55.42
Female	37	44.57
Descent (n=83)		
Asian	15	18.07
African-American	7	8.43
Caucasian	5	6.02
Hispanic	1	1.20
Information not available	55	66.26
HLA-B58* haplotype (n=83)		
Positive	16	19.27
Negative	3	3.61
Information not available	64	77.10
Comorbidities (n=83)		
0-1	22	26.50
2	17	20.48
3 or more	24	28.91
Information not available	20	24.09
Reason for allopurinol use		
Asymptomatic hyperuricemia	36	43.37
Gout	30	36.14
Leukemia	2	2.40
Information not available	15	18.07

Latency Period - Clinical Presentation - Organ Involvement

The latency period from drug initiation to the onset of symptoms ranged from one to 480 weeks (median: 3.71 weeks, IQR: 2.68-5.00 weeks), whereas the daily dosage was documented in 43 cases and ranged from 100 to 1000 mg. Most common symptoms included rash (78 cases, 93.7%), fever (66 cases, 72.5%), lymphadenopathy (29 cases, 34.93%), and facial edema (28 cases, 33.73%). Most common organs affected included the liver, kidneys, lungs, heart, and pancreatobiliary tract. Notably, mucosal involvement (oral, conjunctival, or genital) was reported in 26 cases. A comprehensive summary of the patients' symptomatology is presented in Table 4.

**Table 4:** Reported symptoms, organs involved and diagnostic tests performed in patients with allopurinol-induced DRESS syndrome DRESS: drug reaction with eosinophilia and systemic symptoms; CNS: central nervous system; HHV-6: human herpesvirus 6; EBV: Epstein-Barr virus; CMV: cytomegalovirus; HSV: herpes simplex virus; SARS-CoV-2: severe acute respiratory syndrome coronavirus 2

	No. of patients	Percentage (%)
Presenting symptoms		
Rash	78	93.97
Fever	66	79.51
Lymphadenopathy	29	34.93
Facial edema	28	33.73
Gastrointestinal symptoms	10	12.04
Abdominal pain	5	6.02
Diarrhea	4	4.81
Odynophagia	1	1.20
Dyspnea	8	9.63
Limb edema	8	9.63
Jaundice	5	6.02
Nausea/vomiting	4	4.81
Neurological symptoms (altered mental status, headache, unsteady gait, hemiplegia)	10	12.04
Septic shock	2	2.40
Other (myalgia, weakness, malaise, fatigue, asthenia, decreased appetite, night sweats, pharyngitis, cough, hypotension, oliguria, dysuria)	33	39.75
Organs involved		
Liver	73	87.95
Kidneys	57	68.67
Pancreas-biliary tract	15	18.07
Lungs	8	9.63
Heart	4	4.81
CNS	4	4.81
Endocrine	1	1.20
None	3	3.61
Mucosal involvement (n=83)		
Information not available	39	46.98
Yes	26	31.32
No	18	21.68
Viral reactivation detected		
HHV-6	13	15.66
Other HHV	3	3.61
EBV	6	7.22
CMV	6	7.22
HSV	1	1.20
SARS-CoV-2	1	1.20
Information not available	54	65.06
Biopsy site		
Skin	48	57.83
Another site	12	14.45
Not performed/not reported	27	32.53

Diagnostic Evaluation

Diagnosis relied on a skin biopsy in 48 cases, whereas a biopsy from a different site of involvement, including the renal, liver, lymph node, or bone marrow, was performed in 12 cases [19,25-28]. The most consistent histological patterns included eosinophilic (14 cases) and lymphocytic infiltrates (13 cases), spongiosis (seven cases), interface dermatitis (six cases), eosinophilic infiltration (five cases), keratinocytic necrosis (five cases), and lymphocytoclastic vasculitis (two cases), which provided critical support for clinical diagnosis. Additional methods, including skin patch tests, were performed in four cases, with one positive result, and lymphocyte stimulation tests (LST) or lymphocyte activation tests (LAT) were performed in six cases, yielding four positive and two negative results.

A diagnostic scoring system was employed in 34 cases. The RegiSCAR score was the most frequently applied, being used in 29 cases. Based on the RegiSCAR classification, 19 cases were defined as definite DRESS (score >5), nine cases as probable (score 4-5), and one case as possible (score 2-3). The Japanese consensus criteria for DIHS were utilized in two cases [29,30], while Bocquet’s criteria were applied in one case [31]. The Naranjo probability score for adverse drug reactions was used in two cases [32,33], and the Singer and Wallace criteria, which have since been superseded by the RegiSCAR criteria, were used in one case [34]. Additionally, two cases were diagnosed using pharmacovigilance tools, specifically the Spanish algorithm [35] and the updated French method of imputability [36].

Treatment and Outcomes

Treatment strategy was reported in 80 cases and involved systemic or topical corticosteroids in 76 cases. Antihistamine use was reported in 10 cases, and intravenous immunoglobulin (IVIG) was employed in seven cases [20,37-41]. Immunosuppressive agents such as cyclosporine or tacrolimus were used in four cases [41], whereas antiviral agents were used in two cases [40,42]. N-acetylcysteine was employed as a therapeutic agent in one case [43]. Additional supportive measures, including intravenous fluids, oxygen, antibiotics, vasoactive drugs, or diuretics, were used in 17 cases.

Complications of DRESS syndrome were reported in 49 cases (59.03%). Major complications included acute or chronic renal disease (20 cases), requiring continuous renal replacement therapy (CRRT) in 10 cases. Pre-existing chronic kidney disease was present in 28 patients, many of whom were receiving multiple medications, which may have increased the metabolic burden on the liver and kidneys and hindered drug clearance, thereby amplifying toxicity. Renal involvement itself was more frequent and represented a core manifestation of DRESS rather than a complication, being observed in 57 patients overall. Among these, 24 experienced renal deterioration during the DRESS episode, suggesting acute-on-chronic injury, while 33 developed de novo renal involvement without previously documented kidney disease.

Other reported sequelae included infectious complications, such as septic shock, bacteremia, Pneumocystis jirovecii pneumonia, streptococcal pneumonia, catheter infection, and urinary tract or genital infection (13 cases); liver injury (eight cases); cardiac complications, including cardiac arrest, acute myocardial infarction, myocarditis, pericardial effusion, and acute pulmonary edema (eight cases); respiratory complications, including acute respiratory distress syndrome, pulmonary embolism, and acute respiratory failure (seven cases); neurological complications, including cerebral vasculitis and viral encephalitis (three cases) [44-46]; and hematological complications, including immune thrombocytopenic purpura, disseminated intravascular coagulation, hemophagocytic lymphohistiocytosis, pure red cell aplasia, and acute anemia (six cases) [32,47]. The most frequent minor complication was rash recurrence after rapid corticosteroid tapering, observed in 15 cases. During follow-up, 72 patients were alive, whereas 11 patients died as a result of allopurinol-induced DRESS syndrome. Patient outcome and reported adverse clinical outcomes are listed in Table 5.

**Table 5:** Reported outcomes and complications in patients with allopurinol-induced DRESS syndrome DRESS: drug reaction with eosinophilia and systemic symptoms; CI: confidence interval

	No. of patients	Percentage (%)	95% CI (%)
Total no. of patients (n=83)			
Alive	72	86.74	78–93%
Dead	11	13.25	7-23%
Main complications			
None	34	40.96	30-52%
Renal complications	25	30.12	21–41%
Rash recurrence	15	18.07	11–29%
Liver complications	8	9.63	4-18%
Cardiac complications	8	9.63	4–18%
Cardiac arrest	4	4.81	-
Arrhythmia	1	1.20	-
Myocarditis	1	1.20	-
Pericardial effusion	1	1.20	-
Acute pulmonary edema	1	1.20	-
Respiratory complications	7	8.43	3–17%
Respiratory failure	4	4.81	-
Bronchiolitis	2	2.40	-
Pulmonary embolism	1	1.20	-
Hematologic complications	6	7.22	3–15%
Immune thrombocytopenic purpura (ITP)	1	1.20	-
Disseminated intravascular coagulation	1	1.20	-
Hemophagocytic histiocytosis	2	2.40	-
Pure red cell aplasia	1	1.20	-
Acute anemia	1	1.20	-
Gastrointestinal complications	2	2.40	0.3–8%
Small bowel perforation	1	1.20	-
Erosive gastritis	1	1.20	-
Infectious complications	13	15.66	9–25%
Septic shock - bacteremia	6	7.22	-
Pneumonia	3	3.61	-
Urinary tract infection - genital ulcers	4	4.81	-
Other complications	9	10.84	5 – 19%
Fever	2	2.40	-
Encephalopathy	2	2.40	-
Immune reconstitution syndrome (IRIS)	1	1.20	-
Cerebellar vasculitis	1	1.20	-
Mental deterioration	1	1.20	-
Acute edematous pancreatitis	1	1.20	-
Pseudomembranous conjunctivitis	1	1.20	-

Discussion

Allopurinol is one of the most commonly prescribed urate-lowering agents worldwide, particularly for the management of gout and asymptomatic hyperuricemia [1,48,49]. However, its widespread use has been debated due to the potential risk of life-threatening adverse reactions, including DRESS syndrome. Much of the current knowledge regarding allopurinol-induced DRESS syndrome derives from case reports and data from pharmacovigilance databases worldwide. To our knowledge, this study constitutes the most comprehensive systematic review of DRESS syndrome caused specifically by allopurinol published in the literature to date.

Pathogenesis and Genetic Susceptibility

The pathogenesis of allopurinol-induced DRESS syndrome remains incompletely understood, but current evidence suggests that it involves a complex interplay of immunologic and genetic factors [50]. Central to this process is a CD4⁺ T-cell-mediated immune response that initiates a cascade of cytokine release, ultimately leading to eosinophil activation and systemic inflammation [33]. Oxypurinol, the active metabolite of allopurinol, acts as a hapten by binding to cellular proteins and forming complexes that are presented by HLA molecules to both CD4⁺ and CD8⁺ T cells. This antigen presentation triggers robust T-cell activation, promoting further cytokine release and recruitment of inflammatory cells, including eosinophils, which contribute to tissue damage in organs such as the skin, liver, and kidneys. A key genetic risk factor in this process is the HLA-B*58:01 allele; individuals carrying this allele are more efficient at presenting oxypurinol-related antigens to cytotoxic T cells, greatly increasing the risk of a hypersensitivity reaction [10,51,52]. In addition to this immune activation, reactivation of latent HHV-6 is frequently observed during DRESS episodes [53,54]. This viral reactivation is thought to result from drug-induced immune dysregulation and further amplify systemic inflammation by stimulating T-cell proliferation and activation.

Patient Characteristics and Risk Factors

The median age at diagnosis in our review aligns with previous studies, suggesting that older adults are more susceptible to severe drug reactions, possibly due to the accumulation of comorbidities and polypharmacy [55]. Many patients had underlying renal pathology resulting in chronic kidney disease, as was the case in several patients in our study. This may be explained by impaired renal function leading to reduced clearance of oxypurinol, the active metabolite of allopurinol, which results in its accumulation and an increased risk of hypersensitivity reactions [2]. Underlying renal impairment was another prominent risk factor in our cohort. Renal involvement was a common manifestation of DRESS syndrome, occurring both in patients with pre-existing kidney disease and in those without prior renal impairment.

Furthermore, the overrepresentation of patients of Asian descent in the reported cases corroborates the association between the HLA-B*58:01 haplotype and increased susceptibility to allopurinol-induced DRESS, as confirmed in our study. This haplotype is particularly common in Asian populations and has become a target for pre-prescription genetic screening [56]. The American College of Rheumatology (ACR) currently recommends routine HLA-B*58:01 screening in all patients of Korean, Han Chinese, or Thai descent before initiating allopurinol therapy [56]. 

Clinical Presentation and Organ Involvement

The latency period from drug initiation to the onset of symptoms aligned with the two- to eight-week range reported in the literature. Delayed presentations beyond the typical timeframe were also observed. The clinical manifestations of DRESS syndrome in our study were consistent with previous literature, with rash, fever, lymphadenopathy, and facial edema being the most common presenting symptoms. The rash is typically diffuse and maculopapular, and it can spread across large areas of the body, often accompanied by purpura or other cutaneous eruptions [18]. In cases of systemic involvement, the liver is the most frequently affected organ, presenting with a spectrum of manifestations ranging from asymptomatic elevations in liver function tests to fulminant hepatic failure [43]. The kidneys are the second most commonly involved organ, with clinical presentations varying from mild increases in serum creatinine and proteinuria to permanent renal failure requiring CRRT.

Laboratory examinations vary depending on the organ affected. For instance, hepatic involvement may be associated with hyperbilirubinemia and elevated transaminases [57-59], whereas renal involvement is usually accompanied by elevated creatinine or urea levels or proteinuria [40,60-63]. Hematologic abnormalities may include eosinophilia, leukocytosis, and atypical lymphocytes, especially in cases with multisystem involvement [60]. Our review was consistent with this pattern, with eosinophilia being the most common feature, followed by leukocytosis and the presence of atypical lymphocytes. Additionally, the high incidence of viral reactivation in our study, particularly HHV-6, followed by EBV, CMV, and other viruses, supports the hypothesis of an immune-related mechanism triggered by allopurinol [24,64].

Diagnostic Considerations

The diagnostic process in DRESS syndrome is challenging due to the long latency period, its heterogeneous clinical presentation, and the absence of a single definitive diagnostic test. Skin biopsy remains an invaluable diagnostic tool, offering histopathological evidence to support the clinical diagnosis. In our review, skin biopsy was the most frequently used diagnostic tool. Biopsy from an affected site other than skin, such as liver, lymph node, kidney, or bone marrow, served as an alternative or supportive diagnostic approach.

In cases where histopathological examination is not feasible, alternative diagnostic methods, such as lymphocyte transformation tests (LTT) or skin patch testing, may help establish the diagnosis and identify the offending drug [65-67]. However, negative test results do not exclude the diagnosis, and clinical judgment remains essential [68,69]. Reinitiation of the drug as a method for establishing diagnosis was reported in one case [24] but is generally discouraged due to the high risk of relapse [12]. Several scoring systems, such as the RegiSCAR score and Bocquet’s criteria [70], have been developed for diagnosis; however, these tools often lack specificity due to variability in clinical presentation and their overlap with other severe drug reactions [71]. The different scoring systems used for diagnosing DRESS syndrome are summarized in Table 6.

**Table 6:** Comparison of the diagnostic criteria for DRESS syndrome proposed by Bocquet et al., the Japanese Consensus for DIHS, and the RegiSCAR group Scoring points for each symptom are mentioned in parentheses. According to Bocquet’s criteria, all three are required for establishing a diagnosis. According to the Japanese criteria, the diagnosis is confirmed by the presence of all seven criteria above (typical DIHS) or of five of the seven (atypical DIHS) DRESS: drug reaction with eosinophilia and systemic symptoms; DIHS: drug-induced hypersensitivity syndrome; ALT: alanine aminotransferase; HHV-6: human herpesvirus 6

RegiSCAR criteria [3]	Japanese criteria for DIHS[29]	Bocquet criteria [51]
Rash covering ≥50% of body surface area (0/1)	Maculopapular rash developing >3 weeks after starting a limited number of drugs	Cutaneous drug eruption
Enlarged lymph nodes (0/1)	Lymphadenopathy	Adenopathy >2 cm or hepatitis (liver transaminases >2x normal) or interstitial nephritis/pneumonia/carditis
Fever ≥38.5 °C (0/1)	Fever >38 °C	-
Eosinophilia (0.7-1.499 ×10^9^/L -1 point, *≥* 1.5 ×10^9^/L – 2 points)	Leukocyte abnormalities (≥1: leukocytosis >11 ×10^9^/L, atypical lymphocytosis >5%, eosinophilia >1.5 ×10^9^/L)	Hematologic abnormalities: eosinophilia >1.5 ×10^9^/L or atypical lymphocytes
Eosinophils if lymphocytes <4000 (10–19% -1 point, ≥20% - 2 points)	-	-
Atypical/reactive lymphocytes (0/1)	Liver abnormalities (ALT >100 U/L)	-
Disease duration >15 days (0/1)	Prolonged clinical symptoms after discontinuation of the causative drug	-
Suspicious rash (at least 2 of the following: facial edema, purpura, infiltration, desquamation) (0/1)	HHV-6 reactivation	-
Skin biopsy suggesting alternative diagnosis (0/-1)	-	-
Organ involvement (1 organ involved – 1 point, ≥ 2 organs – 2 points)	-	-
Investigation of ≥3 alternative causes with negative results (1)	-	-

Management and Outcomes

Given the severity of the syndrome and the potential for life-threatening complications, early and aggressive treatment is essential [71]. Management of DRESS syndrome primarily involves the immediate discontinuation of the offending drug, which was uniformly applied across the cases reviewed. Corticosteroids were the most commonly used therapy, and relapse during rapid tapering was reported, highlighting the need for gradual dose reduction over a period of three to six months [65]. A treatment algorithm proposed by Descamps et al. suggests managing DRESS syndrome with topical corticosteroids for mild cases, systemic corticosteroids for more severe cases, and a combination of IVIG with systemic corticosteroids for life-threatening situations, adding antivirals if viral reactivation is confirmed, depending on the severity of symptoms and the extent of organ involvement [65]. Notably, before the definitive diagnosis, a variety of medications and supportive measures were administered, reflecting the rarity of this syndrome and the limited index of early suspicion among practicing clinicians.

The complications associated with DRESS syndrome were varied and severe, including acute or chronic renal and liver injury, acute cardiac events, and respiratory and hematological disorders [68,72]. Renal involvement was common, manifesting as both acute-on-chronic deterioration and de novo kidney injury. Additionally, the high rate of infections observed in our study is likely linked to profound immune activation, characterized by eosinophilia, atypical lymphocytosis, and cytokine release, which is often followed by immune suppression. This immune dysregulation predisposes patients to viral reactivation, bacteremia, and bacterial or fungal infections. The mortality rate of DRESS syndrome in general is reported at around 10%, while the specific mortality rate of allopurinol-induced DRESS syndrome reaches 26.7% [2,73]. The mortality rates and spectrum of complications in our cohort are summarized in Table 6.

Clinical Implications and Future Directions

Close interdisciplinary collaboration between specialties, primarily internists, dermatologists, immunologists, and pathologists, is crucial for early recognition and for optimizing therapeutic outcomes in patients with allopurinol-induced DRESS syndrome. Clinicians should obtain a detailed clinical history that includes the timing of drug initiation, as allopurinol-induced DRESS may occur up to eight weeks after exposure [2,74]. This information can assist in differentiating allopurinol from other potential causative agents, although this timeline does not provide absolute certainty, since there are reports of DRESS occurring several months post-exposure. Additionally, patients should be educated to promptly report any relevant symptoms, such as rash, fever, and lymphadenopathy, even if they do not occur within the eight weeks reported in the literature [75].

The rarity of this syndrome highlights the importance of establishing an international patient registry to compile a larger dataset for thorough investigation. By integrating data from various regions and healthcare environments, the registry could improve our comprehension of disease trends, reveal potential genetic and molecular mechanisms, and ultimately inform the development of targeted therapies [76]. This is essential for enhancing evidence-based practices and creating uniform treatment guidelines for this rare condition [77]. Furthermore, the strong association with the HLA-B*58:01 allele, particularly in Asian populations, suggests that genetic screening should be a routine part of the prescribing process for allopurinol in these patients, as proposed by ACR [78,79]. Some evidence supports HLA-B*58:01 screening individuals older than 60 years of age with chronic kidney disease, given their heightened susceptibility to allopurinol-induced DIHS [37].

Limitations

There are several limitations to our study. The underreporting of certain demographic details, such as patients' ancestry or HLA-B*58:01 status, limits the ability to fully explore the relationship between genetic predisposition and the syndrome’s incidence. In our review, HLA-B*58:01 status was available for only 19 of the 83 cases. The high positivity rate observed among those tested (84.2%) should therefore be interpreted with caution, as genetic testing was likely performed selectively in patients with higher clinical suspicion or in individuals from ancestries known to carry a higher allele frequency. This introduces potential selection bias and limits the generalizability of the positivity rate to the entire cohort.

Additionally, numerous studies did not clearly document the reason for allopurinol prescription, making it difficult to assess whether the prescription was truly necessary and whether the potential risks of allopurinol use were justified. The RegiSCAR score was not consistently reported in all cases. Furthermore, DRESS syndrome may overlap with other systemic drug reactions, such as Stevens-Johnson syndrome (SJS) or toxic epidermal necrolysis (TEN), which present with similar symptoms, particularly involving the skin, thereby complicating the accuracy of case reporting [80-87]. The potential for publication bias, in which more severe or unusual cases are more likely to be reported, also limits the ability to generalize the findings to all patients with DRESS syndrome. Finally, the small sample size and the inclusion of cases published only in English restrict the generalizability of the findings and highlight the need for larger, prospective studies to validate these results.

## Conclusions

Allopurinol-induced DRESS syndrome is a serious and potentially fatal adverse drug reaction that requires a high index of clinical suspicion and a thorough review of the patient’s drug history. Early diagnosis, prompt discontinuation of the offending drug, and initiation of corticosteroids with gradual tapering are crucial for reducing the morbidity and mortality associated with this syndrome. The development and implementation of more standardized diagnostic criteria are also essential for the timely and accurate diagnosis of DRESS syndrome. Furthermore, careful patient selection is critical before initiating allopurinol therapy, particularly for asymptomatic hyperuricemia in populations at higher risk of severe adverse reactions.

## Appendices

Appendix: detailed database search strategy

1) PubMed Search Strategy

Search performed using the following query:

(allopurinol) AND ("DRESS syndrome" OR "Drug Reaction with Eosinophilia and Systemic Symptoms" OR "Drug Induced Hypersensitivity Syndrome" OR "DIHS")

Filters applied:

Publication dates: January 1997 - December 2024

Language: English

Sorted by publication date

2) Scopus Search Strategy

Search performed using equivalent adapted terms:

TITLE-ABS-KEY (allopurinol AND ("DRESS syndrome" OR "Drug Reaction with Eosinophilia and Systemic Symptoms" OR "Drug Induced Hypersensitivity Syndrome" OR DIHS))

Filters applied:

Publication years: 1997-2024

Language: English

Table 7 presents the quality assessment of included case reports using the Joanna Briggs Institute (JBI) critical appraisal tool for case reports.

**Table 7:** Quality assessment of included case reports using the Joanna Briggs Institute (JBI) critical appraisal tool for case reports

Author, Year	Were patient’s demographic characteristics clearly described?	Was the patient’s history clearly described and presented as a timeline?	Was the current clinical condition of the patient on presentation clearly described?	Were diagnostic tests or assessment methods and the results clearly described?	Was the intervention(s) or treatment procedure(s) clearly described?	Was the post-intervention clinical condition clearly described?	Were adverse events (harms) or unanticipated events identified and described?	Does the case report provide takeaway lessons?	Score (out of 8)
Kalfoutzou, 2024 [3]	✓	✓	✓	✗	✓	✓	✓	✓	7
Swaminathan, 2024 [85]	✓	✓	✓	✓	✓	✓	✓	✓	8
Rotulo, 2024 [21]	✓	✓	✓	✓	✓	✓	✗	✓	7
Abusuliman, 2024 [14]	✓	✓	✓	✓	✗	✓	✗	✓	6
Setoyama, 2024 [62]	✓	✓	✓	✓	✓	✓	✓	✓	8
Masior, 2024 [40]	✓	✓	✓	✓	✗	✓	✗	✓	6
Martinez, 2023 [80]	✓	✓	✓	✓	✓	✓	✓	✓	8
Caliz, 2024 [6]	✓	✓	✓	✓	✓	✓	✓	✓	8
Caliz, 2024 [6]	✓	✓	✓	✓	✓	✓	✗	✓	7
Caliz, 2024 [6]	✓	✓	✓	✓	✓	✓	✓	✓	8
Lucas, 2022 [12]	✓	✓	✓	✓	✓	✓	✓	✓	8
Lafkih, 2022 [5]	✔	✔	✔	✔	✔	✔	✔	✔	8
Ben Salha, 2021 [87]	✔	✔	✔	✔	✔	✔	✔	✔	8
Ikediobi, 2021 [37]	✔	✔	✔	✔	✔	✔	✔	✘	7
Ikediobi, 2021 [37]	✔	✔	✔	✔	✔	✔	✔	✔	8
Myers, 2020 [50]	✔	✔	✔	✔	✔	✔	✔	✘	7
Hakim, 2020 [43]	✔	✔	✔	✔	✔	✔	✔	✔	8
Kang SY, 2020 [76]	✔	✔	✔	✔	✔	✔	✘	✔	7
Aatif, 2018 [11]	✔	✔	✔	✔	✔	✔	✔	✔	8
Bayan, 2018 [71]	✔	✔	✔	✔	✔	✔	✔	✔	8
Batista, 2021 [67]	✔	✔	✔	✔	✔	✔	✔	✔	8
Waseem, 2017 [57]	✔	✔	✔	✔	✔	✔	✔	✔	8
Casagranda, 2017 [81]	✔	✔	✔	✔	✔	✔	✘	✔	7
Kim, 2017 [66]	✔	✔	✔	✔	✔	✔	✔	✔	8
Kim, 2017 [66]	✔	✔	✔	✔	✔	✔	✔	✔	8
Kim, 2017 [66]	✔	✔	✔	✔	✔	✔	✔	✔	8
Taghvaye, 2017 [15]	✔	✔	✔	✔	✔	✔	✔	✔	8
Borok, 2016 [26]	✔	✔	✔	✔	✔	✔	✔	✔	8
Solak, 2016 [63]	✔	✔	✔	✔	✔	✔	✔	✔	8
Kato, 2016 [39]	✔	✔	✔	✔	✔	✔	✔	✔	8
Turney, 2016 [82]	✔	✔	✔	✔	✔	✔	✔	✔	8
Mancano, 2015 [59]	✔	✔	✔	✔	✔	✔	✔	✔	8
Civardi, 2015 [64]	✔	✔	✔	✔	✔	✔	✔	✔	8
Lim, 2014 [44]	✔	✔	✔	✔	✔	✔	✔	✔	8
Su, 2014 [83]	✔	✔	✔	✔	✔	✔	✔	✔	8
Choi, 2014 [58]	✔	✔	✔	✔	✔	✔	✔	✔	8
Thankacen, 2015 [38]	✔	✔	✔	✔	✔	✔	✔	✔	8
Sola, 2013 [45]	✔	✔	✔	✔	✔	✔	✔	✔	8
Gallo Puelles, 2013 [4]	✔	✔	✔	✔	✔	✔	✔	✔	8
Lim, 2013 [53]	✔	✔	✔	✔	✔	✔	✔	✔	8
Botelho, 2012 [60]	✔	✔	✔	✔	✔	✔	✘	✔	7
Botelho, 2012 [60]	✔	✔	✔	✔	✔	✔	✔	✔	8
Sekine, 2012 [29]	✔	✔	✔	✔	✔	✔	✘	✔	7
Yaylaci, 2012 [75]	✔	✔	✔	✔	✔	✔	✘	✔	7
Hassan, 2011 [52]	✔	✔	✔	✔	✔	✔	✔	✔	8
Cardoso, 2011 [28]	✘	✔	✔	✔	✔	✔	✔	✔	7
Hamaguchi, 2021 [42]	✔	✔	✔	✔	✔	✔	✔	✔	8
Cooksley, 2010 [2]	✔	✔	✔	✔	✔	✔	✔	✔	8
Dewan, 2009 [20]	✔	✔	✔	✔	✔	✔	✔	✔	8
Calogiuri, 2009 [68]	✔	✔	✔	✔	✔	✔	✔	✔	8
Sackesen, 2008 [22]	✔	✔	✔	✔	✔	✔	✔	✔	8
Shalom, 2018 [48]	✔	✔	✔	✔	✔	✔	✔	✔	8
Suzuki, 2014 [24]	✔	✔	✔	✔	✔	✔	✔	✔	8
Chao, 2018 [32]	✔	✔	✔	✔	✔	✔	✔	✔	8
Descamps, 2003 [13]	✔	✔	✔	✔	✔	✔	✔	✔	8
El Hadj, 2022 [23]	✘	✔	✔	✔	✔	✔	✔	✔	7
Sevinc, 2019 [74]	✔	✔	✔	✔	✔	✔	✔	✔	8
Earlia, 2024 [17]	✔	✔	✔	✔	✔	✔	✔	✔	8
Engell, 2013 [54]	✔	✔	✔	✔	✔	✔	✔	✔	8
Vilchez-Sanchez, 2022 [35]	✔	✔	✔	✔	✔	✔	✔	✔	8
Ponzo, 2021 [25]	✔	✔	✔	✔	✔	✔	✔	✔	8
Mugwagwa, 2021 [51]	✔	✔	✔	✔	✔	✔	✔	✔	8
Marques, 2020 [47]	✔	✔	✔	✔	✔	✔	✔	✔	8
Xiao, 2022 [30]	✔	✔	✔	✔	✔	✔	✔	✔	8
Quach, 2022 [56]	✔	✔	✔	✔	✔	✔	✔	✔	8
Salem, 2015 [33]	✔	✔	✔	✔	✔	✔	✔	✔	8
Teo, 2011 [84]	✔	✔	✔	✔	✔	✔	✔	✔	8
Fathallah, 2010 [34]	✔	✔	✔	✔	✔	✔	✔	✔	8
Kumar, 2019 [73]	✔	✔	✔	✔	✔	✔	✔	✔	8
Ibzinski, 2023 [61]	✔	✔	✔	✔	✔	✔	✔	✔	8
Souilem, 2023 [36]	✓	✓	✓	✓	✓	✓	✓	✓	8
Palacian, 2017 [77]	✓	✓	✓	✓	✓	✓	✓	✓	8
Neki, 2017 [7]	✓	✓	✓	✓	✓	✓	✓	✓	8
Díaz, 2022 [41]	✓	✓	✓	✓	✓	✓	✓	✓	8
Diaz, 2022 [41]	✔	✔	✔	✔	✔	✔	✔	✔	8
Diaz, 2022 [41]	✔	✔	✔	✔	✔	✔	✔	✔	8
Diaz, 2022 [41]	✔	✔	✔	✔	✔	✔	✔	✔	8
Karthika, 2021 [8]	✘	✔	✔	✔	✔	✔	✔	✔	7
Masaki, 2003 [46]	✔	✔	✔	✔	✔	✔	✘	✔	7
Guttierez-Macías, 2005 [49]	✘	✔	✔	✔	✔	✔	✔	✔	7
Elias, 2021 [16]	✘	✔	✔	✔	✔	✔	✔	✔	7
El Anzi, 2019 [31]	✘	✔	✔	✔	✔	✔	✔	✔	7
Bouomrani, 2019 [55]	✘	✔	✔	✔	✔	✔	✔	✔	7
