# Correction: The Hidden Perils of Allopurinol: A Systematic Review of Allopurinol-Induced DRESS (Drug Reaction With Eosinophilia and Systemic Symptoms) Syndrome

**DOI:** 10.7759/cureus.c416

**Published:** 2026-03-20

**Authors:** Areti Kalfoutzou, Christos Piperis, Pantelis Petroulakis, Adam Mylonakis

**Affiliations:** 1 Department of Medical Oncology, 251 Air Force General Hospital, Athens, GRC; 2 Department of Cardiology, Gennimatas General Hospital, Athens, GRC; 3 Second Department of Internal Medicine, 251 Air Force General Hospital, Athens, GRC; 4 First Department of Surgery, Laikon General Hospital, National and Kapodistrian University of Athens, Athens, GRC

This article has been corrected to fix a typo in the PRISMA diagram along with missing reference citations in Table 7 in the Appendix. The Materials and Methods section has been revised to state that "Additional studies were identified by searching the reference lists of relevant articles (n = 20), bringing the total to 308."

